# Sexual obsessions and suicidal behaviors in patients with mood disorders, panic disorder and schizophrenia

**DOI:** 10.1186/1744-859X-11-27

**Published:** 2012-10-30

**Authors:** Liliana Dell’Osso, Giulia Casu, Marina Carlini, Ciro Conversano, Paola Gremigni, Claudia Carmassi

**Affiliations:** 1 Department of Clinical and Experimental Medicine, University of Pisa, Via Roma 67, 56127, Pisa, Italy; 2 Department of Psychology, University of Bologna, Bologna, Italy; 3 Azienda Sanitaria Locale Massa, Pisa, Italy

**Keywords:** Sexual obsessions, Suicidal ideation, Suicidal plans, Suicidal attempt, Mood disorders, Panic disorder, Schizophrenia

## Abstract

**Background:**

The topic of sexual obsessions as a psychiatric symptom has not been well investigated. The aim of this study was twofold: 1) to explore the presence of sexual obsessions in patients with mood disorders (*n*=156), panic disorder (*n*=54) and schizophrenia (*n*=79), with respect to non-psychiatric subjects (*n*=100); 2) to investigate the relationship between sexual obsessions and suicidal behaviors, taking into account socio-demographic variables ad mental disorders.

**Methods:**

289 psychiatric patients with mood disorders, panic disorder or schizophrenia, were recruited at the Italian University departments of psychiatry along with 100 non-psychiatric subjects, who presented for a routine eye exam at the ophthalmology department of the same Universities. The assessments included: the Structured Clinical Interview for DSM-IV-TR, the Brief Psychiatric Rating Scale (BPRS), the Obsessive-Compulsive Spectrum Self-Report (OBS-SR), for sexual obsession, and the Mood Spectrum-Self Report lifetime version (MOODS-SR). Suicidality was assessed by means of 6 items of the MOODS-SR.

**Results:**

Sexual obsessions were more frequent in schizophrenia (54.4%), followed by mood disorders (35.9%). Among schizophrenia patients, males reported more sexual obsessions than females (*P*<0.01). Subjects who were more likely to report suicidal behaviors (suicidal ideation, plans and attempts) were female (adjusted OR=1.99), patients with mental disorders, specifically mood disorders (adjusted OR=11.5), schizophrenia (adjusted OR=3.7) or panic disorder (adjusted OR=2.9), and subjects who reported lifetime sexual obsessions (adjusted OR= 3.6). Sexual obsessions remained independently associated with all aspects of suicidal behaviors. Age, education, marital and employment status were not related to suicidal behaviors.

**Conclusions:**

Special attention should be given to investigate and establish effective strategies of treatment for sexual obsessions, especially those with comorbid mood disorders or schizophrenia.

## Introduction

Sexual obsessions are shown to be included onto the “unacceptable/forbidden thoughts” category [1,2] and may comprise ego-dystonic, intrusive, recurrent and persistent thoughts, images or concerns about sexual matters that do not usually prompt sexual behavior. Thus, sexual obsessions can be considered a form of obsessive compulsive disorder (OCD) themselves [3].

The latent symptom structure of OCD was best defined by four specific dimensions: (a) obsessions and checking (e.g., aggressive, sexual, religious, and somatic obsessions and checking compulsions); (b) symmetry and ordering (e.g., symmetry and exactness obsessions, repeating, counting, and arranging compulsions); (c) contamination and cleaning (e.g., contamination obsessions and cleaning/washing compulsions); and (d) hoarding [2]. In patients with OCD, obsessions and checking were found to be more diffuse (92.78%) and associated with a greater overall functional and lifestyle impairment than other categories of symptoms [4].

In this study, we focused only on sexual obsessions, since the severity of obsessions, but not compulsions, was found to be related to impairment across several domains of life [5,6].

The literal content of sexual obsessions may resemble other types of iterative sexual ideation, as seen in paraphilia, post-traumatic stress disorder, and normal sexual fantasy of the general population. Intrusive thoughts are frequently reported by nonclinical samples of adults (80%–88%) [7]. However, most people are able to dismiss them, whereas clinical sexual obsessions are perceived as personally significant, upsetting, and relentlessly recurrent [8]. Patients who experience such a type of “pure obsessions” (i.e., obsessions that are often characterized by the absence of overt compulsions) appraise intrusive thoughts as dangerous and overly important and, thus, struggle to control their thoughts [9]. Therefore, these patients feel greater distress about unacceptable thoughts than nonclinical populations in terms of the extent of distress, frustration, time lost, and impairment [10].

Sexual obsessions are common symptoms among OCD patients, with prevalence of 13.3-24.9%, [11] and 30.2% in combination with religious obsessions [12]. Hasler et al. [13] found that people with certain sets of OCD symptoms were more like to have certain comorbidities than others. In particular, about 82.1% of OCD patients with sexual obsessions was found to have a comorbid Axis I diagnosis [11]. OCD and depressed patients were found to have in common some dysfunctional appraisals about their most disturbing obsessions: guilt, unacceptability, likelihood thought would come true, danger, and responsibility for having the intrusive thoughts [14]. Other studies have related bipolar disorder to this cluster of symptoms [15] highlighting the need of further studies if we consider that these patients, besides depressed ones, often report sexual dysfunctions and these are associated with increased suicidality [16]. Although these are not uncommon symptoms, the topic of sexual obsessions as a psychiatric symptom and the presence of sexual obsessions within patients with a primary psychiatric diagnosis other than OCD have not been well researched.

In schizophrenia, obsessive compulsive symptoms were reported by 10% [17] to 26.7% of patients, the frequent obsessions being that of contamination, sexual and aggressive thoughts [18]. Although OCD co-occurs in a substantial proportion of schizophrenia patients [19], the rate of occurrence and the clinical effect of sexual obsessions have been scarcely investigated in these patients. Even though the rate of sexual obsessions was not investigated, 23% of patients with a diagnosis of panic disorder were also found to have general obsessive compulsive symptoms [20].

Gender is a relevant factor that should be taken into account when evaluating patients with obsessive symptoms, as a significantly higher frequency of sexual obsessions was observed in males than in females [21].

People with OCD feel a great distress about unacceptable thoughts, to the point that some studies found an association between obsessive compulsive spectrum and suicidality [22,23]. Suicide is defined as the act of intentionally ending one's own life. Its immediate precursors are suicidal behaviors that include suicidal ideation, plans, and attempts [24]. Suicidal ideation consists of thoughts about suicide, which varies greatly from a desire to be dead (passive) to a formulation of intent (active). Suicidal plans refer to making detailed plans to commit suicide, without the suicidal act itself. A suicide attempt consists of a failed act of suicide. The estimated lifetime prevalence of suicidal ideation, plan and attempt in a large overall cross-national sample (N = 84 850) was 9.2%, 3.1% and 2.7%, respectively [24].

Recently, the severity of obsessive compulsive symptomatology, the presence of major depression and aggressive obsessions, and high levels of anxiety or hopelessness have been suggested that could lead patients to consider or attempt suicide to escape from their distressing symptoms [25].

Suicidal behaviors were found to be more common in patients with mood disorders than in other psychiatric disorders, followed by patients with anxiety disorders and impulse-control disorders [24]. Suicidal behaviors are also a frequent complication of schizophrenia with prevalence ranging between 20% and 40% [26-28]. Nevertheless, features of suicidality in schizophrenia patients remain poorly understood [29].

In summary, a large literature indicates an association of OCD and other psychiatric disorders with suicidal behaviors. A much less extensive but consistent literature indicates comorbidities between OCD and other psychiatric disorders. Nevertheless, the influence of subtypes of OCD, such as sexual obsessions, on suicidal behaviors, in patients with a psychiatric diagnosis, was scarcely investigated. A recent study [23] is the only one published article addressing this issue. Results indicated that 36% of patients with OCD reported lifetime suicidal thoughts, 20% had made suicidal plans and 11% had already attempted suicide. In this sample, variables associated with lifetime suicidal thoughts and plans were socio-demographic variables (lower social class, not having children, and religious practice), comorbid mental disorders (major depression, posttraumatic stress disorder, substance use, and impulse-control disorders) and sexual/religious obsessions [23]. Suicide attempts were predicted only by mental disorders.

The purpose of this study was twofold:

1) To estimate the lifetime presence of sexual obsessions and suicidal behaviors in patients with primary diagnoses of mood disorders (major depressive, dysthymic, and bipolar disorders), panic disorder, and schizophrenia, and in a sample of subjects without any psychiatric disorder;

2) To investigate the possible effect of socio-demographic factors, mental disorders and sexual obsessions on suicidal behaviors.

## Method

### Participants

A total of 389 subjects participated in the study. Among them, 289 were consecutive out- and in-patients presenting for treatment at various University departments of psychiatry in north, central and south Italy. New and continuing patients of both genders, between 18 and 60 years of age, who met the DSM-IV-TR criteria for psychiatric disorders, were considered eligible for this study. Exclusion criteria were severe medical illness, neurologic diseases, substance abuse in the month preceding the assessment, and inability to participate in the study due to the severity of the psychiatric symptoms. Of patients included in the study, 156 had a primary diagnosis of mood disorder (major depression, dysthymia, or bipolar disorder), 54 had panic disorder, and 79 schizophrenia. All patients were taking psychotropic medications at the time of assessment.

A sample of non-psychiatric subjects of both genders, aged 18–60 years was also recruited among patients presenting for a routine eye exam at the ophthalmology department of the universities participating in the study. Exclusion criteria were the same that for psychiatric patients. In addition, subjects were also excluded if they met the DSM-IV-TR criteria for psychiatric disorders or had a history of psychiatric disorders, so 100 subjects formed this sample.

The Ethic Committee of the coordinating center (Pisa University Hospital) approved all recruitment and assessment procedures. Eligible subjects provided written informed consent after receiving a complete description of the study. Independent researchers, not members of the therapeutic team, discussed the study with patients and solicited their participation. Refusals to participate in the study did not exceed 5% of subjects.

### Measures

Psychiatric condition was assessed using the Structured Clinical Interview for DSM-IV-TR [30] by psychiatrists or psychiatry residents who were trained and certified in the use of the study instruments.

The severity of current psychiatric symptoms was rated with the Brief Psychiatric Rating Scale [31].

Sexual obsessions were assessed using a self-report scale derived from the Obsessive-Compulsive Spectrum Self-Report (OBS-SR) [32]. This scale included 5 items, coded as present/absent, which asked patients if they have ever felt preoccupied with unwanted and intrusive thoughts about sexual identity, sexual performance, scenes of sexual intercourse or unusual sexual activities, sins, and the impulse to look at someone's crotch or touch other people's genitals. The total score can range from 0 to 5 (from none to a maximum number of five sexual obsessions). The scale reliability in this study exceeded the recommended 0.70 value [33], with Kuder-Richardson Coefficient, K-R 20 = 0.75.

Suicidal behaviors were assessed with 5 items derived from the Mood Spectrum-Self Report (MOODS-SR) lifetime version [34]. The MOODS-SR is an instrument developed and validated by an international collaborative group of clinicians and researchers under the name of the Spectrum Project, which is aimed at assessing lifetime mood spectrum symptoms [35,36]. All MOODS-SR items, including those exploring suicidality, are answered in a 'yes/no' dichotomous format. Three items explore suicidal ideation, asking whether the subject had ever experienced periods of 3 to 5 days or more when he or she thought that life is not worth living, wished he/she would not wake up in the morning or would die in an accident or from something like a heart attack or a stroke, and want to die or hurt him/herself. Answers were summed up and then dichotomized to minimize effects of extreme values. Suicidal plan was assessed by asking if the individual has ever made a detailed plan to kill him/herself. Suicide attempt was assessed by asking whether the subject actually committed at list one lifetime suicide attempt.

Socio-demographic data were collected with regard to age, gender, education, employment situation and marital status. Education was categorized in low (<8 years of school), medium (high school), and high (university degree). Employment condition was dichotomized in employed *vs*. unemployed/retired/housewife. Marital status was dichotomized in single *vs.* non-single.

### Statistical analyses

Demographic characteristics of samples were compared using the χ^2^ tests and analysis of variance (ANOVA) in the case of age. Cross-tabulations with χ^2^ tests were also used to estimate lifetime prevalence of sexual obsessions and suicidal behaviors (ideation, plans and attempts) between groups, taking into consideration the effect of gender. Association of the same variables with age was calculated with point biserial coefficient of correlation.

Logistic regression analyses were performed for all respondents that had ever had the outcomes of interest using a hierarchical 3-step approach. Socio-demographic variables were first entered into the equation, followed by mental disorders and then by sexual obsessions. Multivariate significance was evaluated using Wald χ^2^ test, also comparing models at each step to see whether the variables introduces in the subsequent step provided an improvement (χ^2^ test Δ value) in predictive power. Logistic regression coefficients were transformed in odds ratios (ORs) and 95% confidence intervals (CIs) were also reported. Statistical significance was evaluated using two-tailed 0.05-level tests.

All statistical analyses were performed using the IBM SPSS Statistic 19.0 software package.

## Results

### Participants’ characteristics

Males were more frequent among patients with schizophrenia (69.6%) than among the other groups (24.1-36%). Non-psychiatric subjects were younger (mean age 30.5 years) than patients (mean age between 36.3 and 39.6 years) and had a higher level of formal education (50% with high education) than the other groups (8.9-18.5%). Being single was more frequent among schizophrenics (86.1%) and non-psychiatric subjects (77%) than among mood and panic disorder patients (51.3% and 48.1%, respectively). Being employed *vs*. unemployed was not significantly different between groups. Demographic characteristics of participants and results of tests of difference are presented in Table 1.

**Table 1:** Comparison between subjects on demographic and clinical variables

**Variables**	**Mood disorders (*n*= 156)*n*(%)**	**Panic disorder (*n*= 54)*n*(%)**	**Schizophrenia (*n*= 79)*n*(%)**	**Non-psychiatric*(n*= 100)*n*(%)**	**Test of difference**
Male gender^a^	58 (37.2)	13 (24.1)	55 (69.6)	36 (36)	χ^2^_(3)_ = 34.89*
Education level^b^					χ^2^_(6)_ = 61.62*
Low	74 (47.4)	15 (27.8)	41 (51.9)	5 (5)	
Medium	55 (35.3)	29 (53.7)	31 (39.2)	45 (45)	
High	27 (17.3)	10 (18.5)	7 (8.9)	50 (50)	
Marital status: Single^c^	80 (51.3)	26 (48.1)	68 (86.1)	77 (77)	χ^2^_(3)_ = 83.03*
Job status: Employed	73 (46.8)	33 (61.1)	37 (46.8)	39 (39)	χ^2^_(3)_ = 6.87
Sexual obsessions^d^	56 (35.9)	11 (20.4)	43 (54.4)	11 (11)	χ^2^_(3)_ = 43.49^*^
Suicidal ideation^e^	106 (67.9)	20 (37)	35 (44.3)	16 (16)	χ^2^_(3)_ = 68.42*
Suicidal plan^f^	107 (68.6)	21 (38.9)	35 (44.3)	16 (16)	χ^2^_(3)_ = 69.47*
Suicide attempt^h^	42 (26.9)	6 (11.1)	14 (17.7)	3 (3)	χ^2^_(3)_ = 26.47*
	Mean ± SD	Mean ± SD	Mean ± SD	Mean ± SD	
Age^h^	36.39 ± 10.27	39.57 ± 11.39	36.28 ± 9.88	30.52 ± 9.06	F_(3,388)_ =15.42*

*P <0.001 for Pearson chi-square or ANOVA.

Pairwise comparisons at *P* < 0.01:

^a^ Schizophrenia > Mood disorders, Panic disorder, and Non-psychiatric subjects.

^b^ Low Education: Schizophrenia and Mood disorders > Non-psychiatric subjects.

^b^ High Education: Non-psychiatric subjects > Mood disorders, Panic disorder, and Schizophrenia.

^c^ Schizophrenia and Non-psychiatric subjects > Mood disorders and Panic disorder.

^d^ Schizophrenia > Mood disorders, Panic disorder, and Non-psychiatric subjects.

^e f^ Mood disorders > Schizophrenia, Panic disorder, and Non-psychiatric subjects. Schizophrenia > Non-psychiatric subjects.

^g^ Mood disorders > Panic disorder and Non-psychiatric subjects. Schizophrenia > Non-psychiatric subjects.

^h^ Tamhane post-hoc comparisons at *P* <0.01: Schizophrenia, Mood disorders, and Panic disorder > Non-psychiatric subjects.

### Sexual obsessions and suicidal behaviors: prevalence and difference between groups

Descriptive statistics (Table 2) indicated that 20.4-54.4% of patients *vs*. 11.1% of non-psychiatric subjects reported having been disturbed by sexual obsessions. Pairwise comparisons indicated that schizophrenia patients reported sexual obsessions more frequently than the other groups of participants, followed by mood disorders patients (35.9%). Only among non-psychiatric subjects, males reported sexual obsessions more frequently than females.

**Table 2:** Comparisons between genders by groups on sexual obsessions and suicidal behaviors

		**Sexual obsessions**^**a**^***n*(%)**	**Suicidal ideation**^**b**^***n*(%)**	**Suicidal plan**^**c**^***n*(%)**	**Suicide attempt*n*(%)**
Mood disorders	Male (*n* = 58)	26 (44.8)	37 (63.8)	37 (63.8)	12 (20.7)
	Female (*n* = 98)	30 (30.6)	69 (70.4)	70 (71.4)	30 (30.6)
	Total (*n* = 156)	56 (35.9)	106 (67.9)	107 (68.6)	42 (26.9)
Panic disorders	Male (*n* = 13)	3 (23.1)	5 (38.4)	5 (38.4)	1 (7.7)
	Female (*n* = 41)	8 (19.5)	15 (36.6)	16 (39)	5 (12.2)
	Total (*n* = 54)	11 (20.4)	20 (37)	21 (38.9)	6 (11.1)
Schizophrenia	Male (*n* = 55)	31 (56.4)	20 (36.4)	20 (36.4)	9 (16.4)
	Female (*n* = 24)	12 (50)	15 (62.5)	15 (62.5)	5 (20.8)
	Total (*n* = 79)	43 (54.4)	35 (44.3)	35 (44.3)	14 (17.7)
Non-psychiatric	Male (*n* = 36)	8 (22.2)	5 (13.9)	5 (13.9)	1 (2.8)
	Female (*n* = 64)	3 (4.7)	11 (17.2)	11 (17.2)	2 (3.1)
	Total (*n* = 100)	11 (11)	16 (16)	16 (16)	3 (3)

^a^ Pearson χ^2^_(1)_ = 15.31, *P* < 0.001. Pairwise comparisons at *P*< 0.01: Non-psychiatric male > female.

^b^ Pairwise comparisons at *P*< 0.01: Schizophrenia female > male.

^c^ Pairwise comparisons at *P*< 0.01: Schizophrenia female > male.

Concerning suicidal behaviors, both suicidal ideation and plans were reported by mood disorders patients (67.9% and 68.6%, respectively) more frequently than by the other groups of patients and non-psychiatric subjects (16%); schizophrenia patients also reported more ideation and plans (44.3%) than non-psychiatric subjects. Mood disorders patients reported more suicide attempts (26.9%) than both panic disorder patients (11.1%) and non-psychiatric subjects (3%), and schizophrenia patients reported more attempts (17.7%) than non-psychiatric subjects.

The frequencies of suicidal ideation and plans almost coincided in this study; thus, these two dimensions were grouped into one in the subsequent analyses.

About age, it was not related to any of the variables of interest (r = 0.40 with sexual obsessions; r_pb_ = 0.07, 0.08, and 0.06 with suicidal ideation, plan and attempt, respectively). Exploring these relations for each group, separately, correlation coefficients varied between −0.18 to 0.19, with *P* >0.05.

### Relation between sexual obsessions and suicidal behaviors

Results of logistic regression analyses showed that gender, mental disorders and sexual obsessions were significantly related to suicidal behaviors in this study. Specifically, being female, having been diagnosed with mood disorders, schizophrenia or panic disorder, and having ever had sexual obsessions is associated with both suicidal ideation/plan and attempt. The ODs of gender were fairly modest in magnitude (adjusted OR = 1.99-2.04), while none of the other socio-demographic variables was related to suicidal behaviors.

The presence of a mental disorder was associated with significantly increased risk of suicidal behaviors. Relations were strongest for mood disorders (adjusted OR=11.5) followed by schizophrenia (adjusted OR=3.7) and panic disorder (adjusted OR=2.9–3.0).

The presence of sexual obsessions was associated with significantly increased risk of suicidal behaviors (adjusted OR=3.59-3.68), even after controlling for socio-demographic characteristics and mental disorders. Results of logistic regressions are presented in Table 3.

**Table 3:** Associations between socio-demographic factors, mental disorders, sexual obsessions and suicidal behaviors

**Factor**	**Suicidal ideation and plan**		**Suicidal attempt**	
	**OR**	**95% CI**	**OR**	**95% CI**
Gender: Female	1.99*	1.19-3.32	2.04*	1.22-3.39
Age	0.98	0.96-1.01	0.98	0.96-1.01
Education^a^:				
Low	0.92	0.48-1.72	0.91	0.44-1.88
High	0.82	0.46-1.46	1.31	0.59-2.17
Job condition: Unemployed	0.89	0.56-1.98	0.89	0.55-1.46
Marital status: Single	1.05	0.55-1.45	1.11	0.59-2.17
Step 1 Wald values	χ^2^ = 10.91_(6)_		χ^2^ = 12.34_(6)_	
Mental diagnosis^b^:				
Mood disorders	11.48**	5.62-20.6	11.51**	5.00-18.3
Panic disorders	2.87*	1.32-6.66	3.02**	1.30-7.03
Shizophrenia	3.66*	1.61-7.71	3.69**	1.57-6.67
Step 2 Wald values	χ^2^ = 78.84_(9)_** Δ = 67.93_(3)_**		χ^2^ = 36.67_(9)_** Δ = 24.32_(3)_**	
Sexual obsessions	3.68**	2.15-6.30	3.59**	2.09-6.01
Step 3 Wald values	χ^2^ = 103.10_(10)_** Δ = 24.26_(1)_**		χ^2^ = 45.25_(10)_** Δ = 8.59_(1)_*	

*P <0.01 and **P <0.001 for logistic regression with Wald χ^2^ test.

Δ = step incremental χ^2^value.

^a^ Reference category: Medium education.

^b^ Reference category: Non-psychiatric subjects.

## Discussion

This paper addresses a critical issue in the relationship between sexual obsessions and psychiatric diagnosis that is quite neglected in the literature. A possible reason for that is the tendency of researchers and clinicians to group sexual obsessions with aggressive and religious obsessions that make it difficult to identify the contribution of each to the individual distress [13,37]. Furthermore, some research categorizes sexual obsessions and compulsivity together with deviant behavior, although deviant individuals experience thoughts, impulses, and behaviors as enjoyable, and not distressing [38]. Another reason may be the tendency, shown by patients, to deny or minimize their sexual obsessions [11].

Results of this study show that sexual obsessions affect more than half of patients with schizophrenia and over a third of those with mood disorders. This finding may be consistent with studies in the literature reporting a high proportion of obsessive compulsive symptoms in depressed patients [11]. About schizophrenia, instead, the presence of obsessive symptoms appears to be more relevant in this study than in the literature (54% *v*s. 10-26%) [17,18]. It may be due to differences in measuring sexual obsessions between studies.

The proportion of sexual obsessions reported by panic disorder patients in this study was similar to that of obsessive compulsive symptoms reported by these patients in the literature (20% *vs.* 23%) [20]. Finally, sexual obsessive symptoms seem to affect a minority of non-psychiatric subjects (11%) as previous observations suggested [39].

About gender, males reported more sexual obsessions than females among non-psychiatric subjects, as expected [21]. Conversely, no difference between genders was found among all groups of patients. This result is congruent with a study indicating that, among patients with sexual obsessions, 53.8% were female [11]. Altogether, there is a need to explore, accurately, the prevalence of sexual obsessions in patients with different psychiatric diagnoses of both genders.

Another finding of this study concerns the prevalence and differences between groups and genders on suicidal behaviors. Patients with mood disorders reported higher suicidal ideation, plan and attempt than the other groups of patients and non-psychiatric subjects, as expected. In fact, the recurrence of suicidal ideation was high across depressive episodes [40-43]. Also, the presence of mixed symptoms such as pseudo-unipolar depression substantially increases the risk of both attempted and committed suicide [43-45]. In a recent study, 62% of patients with psychotic mood disorders and 56% of patients with non-psychotic mood disorders reported suicidal ideation [27].

In this study, schizophrenia patients also reported more suicidal behaviors than non-psychiatric subjects and this rate was in the range of that of other studies (20-40%) [26-28], although in this study suicidal ideation and plans were much more frequent than attempts (44% *vs*. 17%).

There were no differences between genders in almost all study groups except in patients with schizophrenia. Among them, females reported more frequently suicidal behaviors than males, as it was found in the literature for patients with mental disorders and the general population [24].

Another important objective of the present study was to explore the possible effect of sexual obsessions on suicidal behaviors, taking into account also socio-demographic characteristics and different psychiatric diagnoses. Results show that among genders, being female is associated with suicidal behaviors, whereas none of the other socio-demographic factors is related to suicidal behaviors. In a large cross-national sample, risk of each suicidal behavior was found to be significantly related to female gender, younger age, having fewer years of formal education, and ever being married [24]. Nevertheless, the ORs were remarkably modest in magnitude, with the exception of age that decreased as the risk of each suicidal behavior increased. The different results regarding age could be explained by the different composition of the two study samples. The reference group to calculate the ORs in the study by Nock et al. [24] was an age >64 years, whereas in this study the maximum age was 60 years and 50% of cases were about 35 years old. Employment status was unrelated to suicidal behaviors in both studies.

Mental disorders also contribute to the likelihood of reporting lifetime suicidal behaviors, with a substantial contribution coming from mood disorders, followed by schizophrenia, and panic disorder. This result is partially consistent with the results of Nock et al. [24] observing that mood disorders were strongly associated with suicidal behaviors, although in the present study this contribution was stronger (OR = 11.5 *vs.* 3.4-5.9). The contribution of panic disorder in this study was in the range of that of anxiety disorders (a broader category including panic disorder) in Nock et al. [24] study (OR = 2.9-3.0 *vs*. 2.8-4.8). Unfortunately, we cannot compare the contribution of schizophrenia to suicidal behaviors in the two studies, because the cross-national survey excluded this mental disorder. Nevertheless, suicide behaviors are a relevant issue to be studied in schizophrenia as suicide attempt was found to be a predictor of suicide in these patients (OR = 3.66) [46].

Finally, sexual obsessions add a significant contribution to the tested models, almost triplicating the likelihood of a suicidal behavior (ideation, plan or attempt) among all the study samples. This result is consistent with that of a recent study by Torres et al. [23] that found an association between sexual obsessions, although grouped with religious obsessions, and suicidal thoughts and plans in OCD patients. A difference between the two studies concerns the diagnoses of participants: Torres et al. [23] involved a large sample of OCD patients with various comorbidities, while this study involved both patients with a primary diagnosis other than OCD and non-psychiatric subjects.

Some individuals have a great deal of difficulty controlling their frequent, intrusive thoughts about sex and their gender identity. The present study suggests that these symptoms may have relevance characterizing phenotypes respect to suicidality, in patients with psychiatric diagnoses. Our findings, taken together, stress the clinical relevance of sexual obsessions, and raise the question about the possible psychopathological mechanisms able to explain the link between unwanted sexual thoughts and suicide precursors. It can be hypothesized that sexual obsessions can elicit suicidality when their disturbing content assumes a personal meaning so high as to be able to reduce considerably self-esteem, inducing pathological guilt and self-depreciation. Cognitive theories of OCD posit that obsessions and thoughts appraised as important and personally significant are expected to be persistent and highly upsetting [8]. If individuals regard their sexual obsessions as immoral, sinful, or as potential causes of a complete loss of control or horrific actions, they can favor suicide plans as a possible solution to feelings of shame and guilt. Bordelau [47] suggests that suicide may reveal a form of repressed self-hate and appear as a solution to the individual’s problem. This hypothesis would be worth to be tested with further investigation.

### Limitations

Several limitations of this study are necessary to note. First of all, the cross-sectional correlational design does not allow establishing a causal relationship or a temporal sequence between sexual obsessions and suicidal ideation or attempt. A second limitation is the reduced sample size that did not allow taking into consideration a number of subcategories within each mental disorder. This is the case of mood disorders, where we held together patients with major depression, dysthymia and bipolar disorder, following the example of Nock et al. [24]. Another consequence of the reduced sample size was that the study groups were not well-matched on socio-demographic variables.

A potential criticism is that the presence of lifetime suicidal ideation, plans and attempts was assessed retrospectively through self-reporting. Nevertheless, evidence from the literature indicates that self-rating of suicidality contains considerable probative value, hence, it was suggested to be taken as the primary data source [48].

Finally, some clinical issues that were outside the frame of this study would deserve to be investigated in relation to the variables observed here. Among these: the co-occurrence of personality disorders, a history of childhood sexual abuse, and the sexual orientation of participants.

## Conclusions and clinical implications

In conclusion, this study suggests that is necessary to detect, accurately, the presence of sexual obsessions, which are a source of psychological burden that could be associated with suicide ideation or attempt.

Sexual obsessive symptoms are often misunderstood by both clinicians and patients. They may be misdiagnosed as fantasies or wishes by mental health professionals and misinterpreted as anxiety or depressive ruminations by patients. Such misconceptions can result in errors in treatment that may increase symptoms and contribute to develop suicidal ideation [49]. The sensitive nature of unwanted sexual thoughts and the great potential for misdiagnosis make essential that these symptoms are identified and well-understood by clinicians to aid to improve diagnosis and treatment.

In terms of psychotherapeutic intervention, research on OCD suggests that, compared to most other forms (i.e., contamination, checking, etc.), sexual obsessions take longer to treat and response may be less robust [50-52]. Therefore, particular attention should be given to establish effective strategies of treatment for these symptoms.

Future studies should include analyses of symptoms from epidemiological studies and investigation of treatment outcomes, to provide greater insight into these often misunderstood symptoms [53].

## Competing interests

The authors declare that they have no competing interests.

## Author’s contributions

LDO and CCar participated to the conception and design of the study, the interpretation of the data, the draft and critical revision of the article. GC, MC, CCon and PG participated to the interpretation of the data, the draft and critical revision of the article. All authors agreed to be cited as co-authors, accepting the order of authorship, and approved the final version of the manuscript and the manuscript submission to Annals of General Psychiatry.
